# The characteristics of proteome and metabolome associated with contrasting sperm motility in goat seminal plasma

**DOI:** 10.1038/s41598-021-95138-9

**Published:** 2021-07-30

**Authors:** Baoyu Jia, Jiachong Liang, Chunrong Lv, Sameeullah Memon, Yi Fang, Guoquan Wu, Guobo Quan

**Affiliations:** 1 College of Veterinary Medicine, Yunnan Agricultural University, Kunming City, Yunnan Province China; 2 Yunnan Animal Science and Veterinary Institute, Jindian, Panlong County, Kunming City, Yunnan Province China; 3 Yunnan Provincial Engineering Laboratory of Animal Genetic Resource Conservation and Germplasm Enhancement, Jindian, Panlong County, Kunming City, Yunnan Province China; 4 Jilin Provincial Key Laboratory of Grassland Farming, Northeast Institute of Geography and Agoecology, Chinese Academy of Sciences, Changchun City, Jilin Province China

**Keywords:** Biotechnology, Developmental biology

## Abstract

Sperm motility is an index tightly associated with male fertility. A close relationship between seminal plasma and sperm motility has been confirmed. This study was to assess the protein and metabolite profiles of seminal plasma obtained from adult goats with high or low sperm motility using the proteomic and metabolomic strategies. In total, 2098 proteins were found. 449 differentially abundant proteins (DAPs) were identified, and 175 DAPs were enriched in the high motility group. The obtained DAPs primarily exist in cytoplasma and extra-cellular portion. The Gene Ontology enrichment analysis demonstrated the main functional roles of these DAPs in regulating biological process, metabolic process of organic substances, cellular-metabolic process, primary-metabolic process, metabolic process of nitrogen compounds, etc. Additionally, the Kyoto-Encyclopedia of Genes and Genomes (KEGG) analysis revealed that these DAPs were primarily involved in phosphatidylinositol signaling system, salivary secretion, proteasome, apoptosis, mitophagy-animal, etc. Aided by the parallel reaction monitoring technology, the abundance changing pattern of 19 selected DAPs was consistent with that of the corresponding proteins obtained by TMT. A total of 4603 metabolites were identified in seminal plasma. 1857 differential metabolites were found between the high motility group and the low motility group, and 999 metabolites were up-regulated in the high motility group. The KEGG analysis demonstrated the primary involvement of the differential metabolites in metabolic and synthetic activities. In conclusion, we first established the proteome and metabolome databank of goat seminal plasma, detecting some proteins and metabolites which may affect sperm motility. This study will be valuable for understanding mechanisms leading to poor sperm motility.

## Introduction

Artificial insemination (AI) can be definitely thought as one of the earliest and the most extensively utilized assisted reproductive technologies in animal breeding and production^1,2^. Currently, AI has been extensively applied in the dairy industry^3^. In general, the site where semen is deposited greatly influences the fertilization results of oocytes with sperm. Moreover, the number of motile sperm is also tightly associated with the conception rate. It’s known that the features of sheep and goat reproductive tract are different from that of cow, which prevents lots of sperm entering uterus and makes sperm walk a long way to arrive at the oviduct and fertilize with oocytes^4^. So, it is becoming important to select those males that can produce semen with a high quality.

Healthy sperm motility is widely recognized as a core factor which can influence male fertility. Those defectively mobile or immobile sperm are usually unfruitful or sterilized except that some assisted reproductive techniques are used^5^. Some AI centers are currently using wave motion of semen as a main index to choose ejaculates for AI in sheep^6^. Furthermore, mass motility has been confirmed to be closely related to fertility in sheep^7^. However, the root causes leading to poor sperm motility are complicated and have not been determined until now.

Seminal plasma is an important part of semen, which is mainly composed of secretions derived from testicular, epididymis, and secondary sex gland. In seminal plasma, the components generally consist of proteins, ions, and metabolites like nucleosides, lipids, monosaccharides, amino acids, minerals, electrolytes, and also steroid hormones^8^. Previous studies have proved that seminal plasma contributes a significant role in regulation of sperm motility and fertilization^9–11^. Seminal plasma provides metabolic support, and it has a complicated and not well-understood effect on the physiological role of sperm. Additionally, seminal plasma also has certain impacts on the quality of chilled^12^ or frozen-thawed sperm^13,14^.

Some studies investigated the effects of proteins presented in seminal plasma on semen quality. In sheep, some investigators have attempted to study the proteome of seminal plasma^14–16^. In accordance with the report of Rickard et al., variations in freezing resistance of sheep sperm is linked with origin and composition of seminal plasma^14^. In pig, the seminal plasma proteome and the associations between seminal plasma proteins and semen features have been established. Particularly, sperm motility had a strongly positive correlation with lactadherin^11^. In human, the analysis related to the seminal plasma proteome reflects reduced mitochondrial production, acrosome disruption, and DNA fragmentation, with several post-genomic functions associated to these alterations^10^. Similarly, in rabbit, major seminal plasma-derived proteins contribute to the prevention of lipid peroxide radical damage and oxidative stress, membrane stability, sperm membrane transport and temperature control. Additionally, sperm motility had positive correlations with growth factor beta-nerve and cysteine rich secreted protein-1. However, a significantly negative association of sperm motility with galectin-1 existed^9^.

Since metabolites were end products of metabolic pathways, which also play important role in sperm physiology, such as motility, energy metabolism, and metabolic activity regulations^17^. In bull, some metabolites in seminal plasma, including 2-oxoglutaric acid or fructose, can be used to evaluate their fertility as possible biomarkers^18^. Hamamah et al. (1993) studied fertile and infertile male seminal plasma using 1 H nuclear-magnetic resonance spectra (NMR), finding important variations in concentrations of glyceryl phosphorylcholine citrate and lactate among azoospermic and oligoasthenozoospermic patients^19^. More recently, Velho et al. (2018) identified 63 metabolites in bull seminal plasma, including 21 amino acids from bull with distinct field fertility ratings, demonstrating the different metabolite patterns between the low-fertility and high-fertility bulls^20^.

Currently, the application of AI in the goat industry is not popular as compared to the other stocks, such as cow or sheep. Moreover, the effect of seminal plasma on sperm motility in goats until now has not been clarified. In this study, we attempted to examine the variation of proteome and metabolome in goat seminal plasma associated with sperm motility using the high throughput technologies. This study will enrich our omics information related to goat semen. Furthermore, some obtained specific proteins or metabolites may be used as biomarkers for assessing the quality of goat semen and predicting of male fertility.

## Results

### Sperm quality assessment

**Figure 1:**
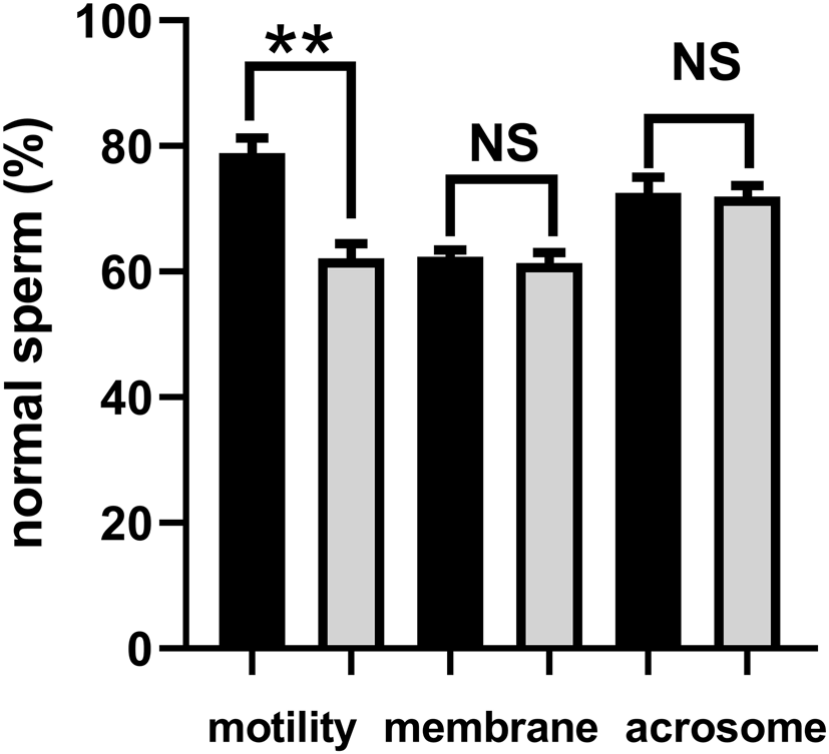
The motility, plasma membrane and acrosome integrity of goat sperm for the metabolite analysis. The black column represented the high motility group, and the grey column represented the low motility group. All data were expressed as means ± SEM. “**” represents a significant difference (*P* < 0.01). “NS” represents no difference (*P* > 0.05).

In this study, for the metabolite analysis, twenty goats were equally separated into two groups. As shown in Fig. 1., one group had a high motility (78.85% ± 2.44%). The other one had a low motility (62.16% ± 2.27%). The difference between these two groups was significant (*P* < 0.01). However, no differences were found among these two groups with regards to the plasma membrane and acrosome integrity (*P* > 0.05).

**Figure 2:**
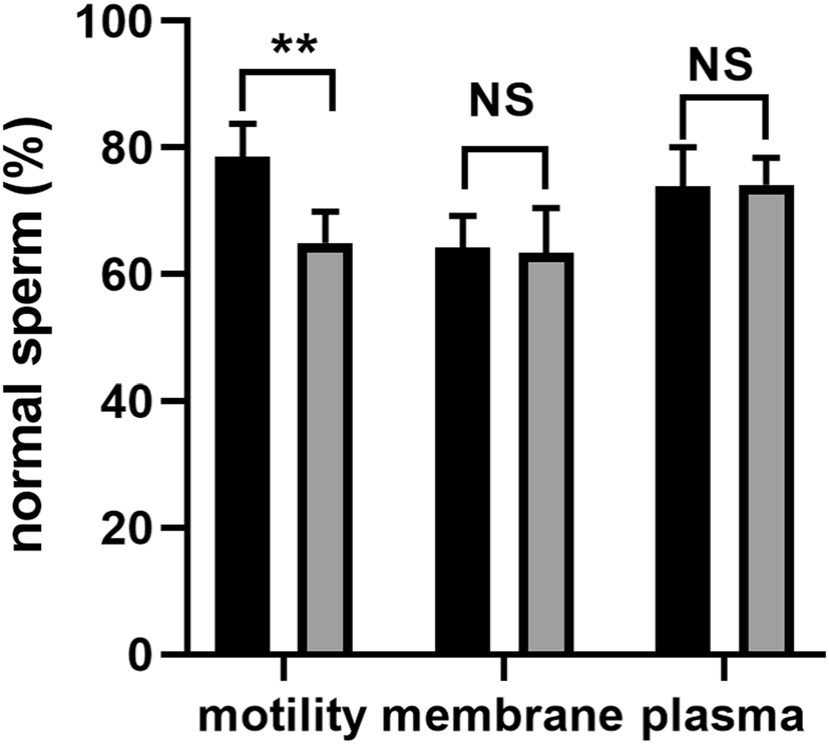
The motility, plasma membrane and acrosome integrity of goat sperm for the proteomic study. The black column represented the high motility group, and the grey column represented the low motility group. All data were expressed as means ± SEM. “**” represents a significant difference (*P* < 0.01). “NS” represents no difference (*P* > 0.05).

For the proteomic analysis, 10 goats were randomly selected from these 20 goats used in this study and equally divided into two groups based on the motility values. The data on sperm motility, membrane integrity, and acrosome integrity were presented in Fig. 2. Similar to Fig. 1., there is significant difference between these two groups (78.56% ± 5.24% vs 64.97% ± 4.87%, *P* < 0.01) in terms of sperm motility. No difference was found regarding membrane and acrosome integrity (*P* > 0.05).

### Identification of proteomic information

A total of 308,933 spectra were obtained after the TMT analysis and searching by Maxquant (v1.5.2.8) in the present study. As shown in Supplementary Fgure S1, the number of the matched spectra was 36,946. Furthermore, we detected 15,724 peptides (14,861 specific peptides) among these spectra, and 2299 proteins including 2098 quantified proteins. The basic information on the protein profile of goat seminal plasma, such as protein accession, protein definition, gene name, *P* value, etc., was revealed in Supplementary Table S1.

### Identification of DAPs

In total, 449 DAPs, with 1.5 folds shift and *P* value of less than 0.05, were identified in goat seminal plasma with high or low motility. The detailed information associated with the identified DAPs, such as protein accession, protein definition, regulated type, *P* value, gene name, etc., was shown in Supplementary Table S2. In comparison with the low motility group, 175 proteins were up-regulated in the high motility group, such as beta-galactosidase, ATP-citrate synthase, 3-phosphoinositide-dependent protein kinase 1-like, trafficking protein particle compound subunit 13 isoform X2, kinesin-like protein, a protein phosphatase inhibitor 2, etc. On the other hand, the abundance of 274 proteins significantly reduced in the high motility group, such as putative adenylate kinase 7, calpain-7-like protein, transmembrane protein 190, fibrous sheath interacting protein 2, angiotensin-converting enzyme, casein kinase II subunit beta, sperm acrosome-associated protein 5, etc.

### Functional classification of DAPs

**Figure 3:**
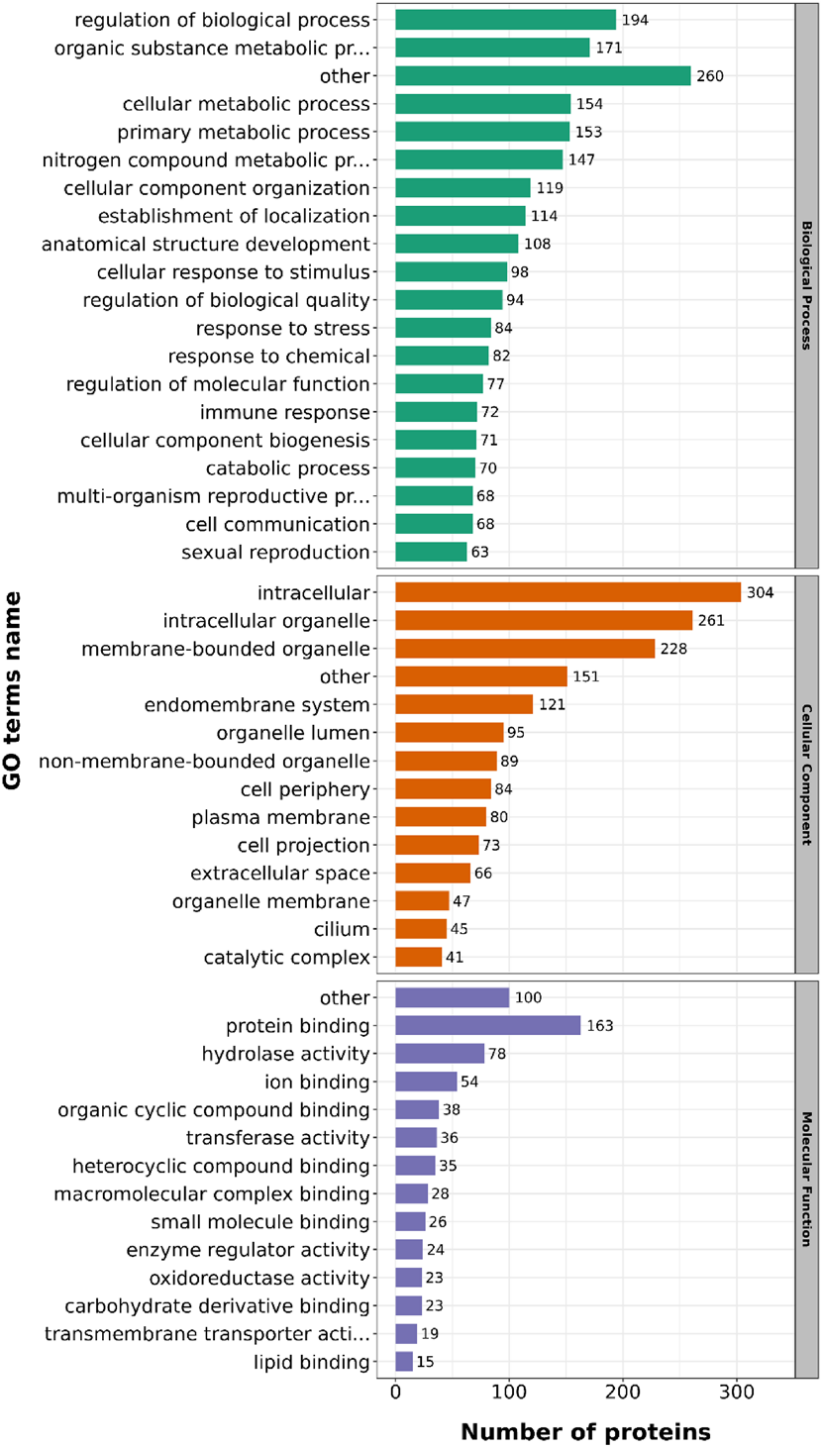
Statistical distribution chart of the identified DAPs based on each GO category. The DAPs were classified into biological process, cellular compartment and molecular function. The bar represents the number of DAPs.

The results of the GO annotation analysis were presented in Fig. 3. Regarding the classification of biological process, the acquired DAPs were significantly enriched in regulating biological processes, organic substance metabolic procedure, cellular metabolic procedure, primary metabolic procedure, nitrogen compound metabolic procedure, cell component organization, localization establishment, etc. Additionally, the acquired DAPs were primarily located in the intracellular region, intracellular organelle, membrane-bound organelle, endomembrane system, organelle lumen, non-membrane-bound organelle, etc., with regard to the GO analysis of cellular component. As concern molecular function, the DAPs were primarily involved in protein binding, hydrolase activities, ion binding, organic cyclic complex binding, transferrase activity, heterocyclic complex binding, etc.

**Figure 4:**
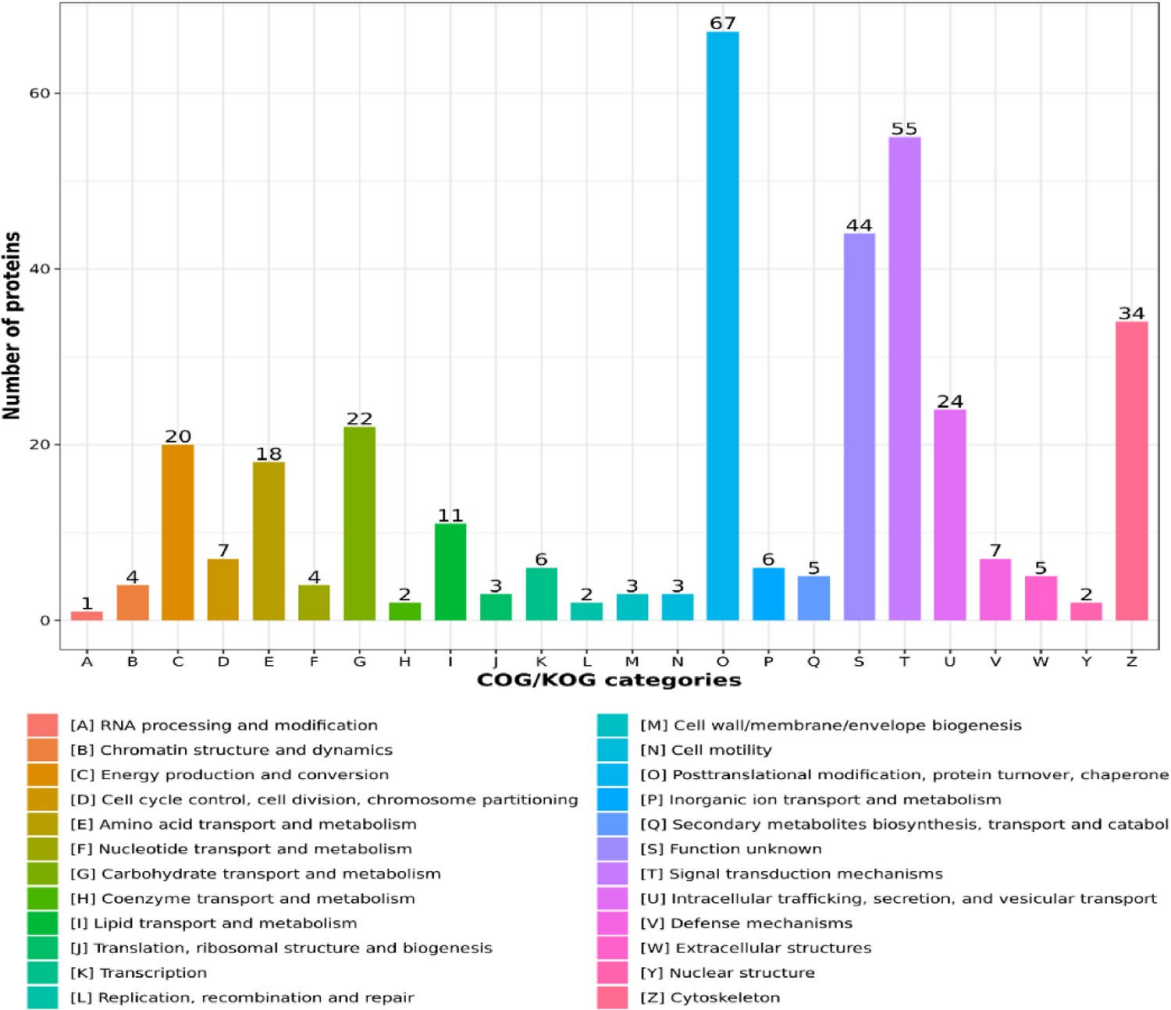
COG/KOG functional classification chart of the identified DAPs. The values on each bar represented the number of proteins that are involved in this function.

Moreover, the COG/KOG functional classification statistics related to the acquired DAPs were presented in Fig. 4. 67 DAPs were found to be involved in post-translational modification, protein turnover, and chaperone. 55 DAPs may have potentially functional roles in the pathways of signal transduction. There were 34 DAPs engaged in cytoskeleton. Additionally, some DAPs were found to function in metabolic activities, including energy manufacturing and adaptation (20), amino acids carrying with metabolism (18), carbohydrates carrying with metabolism (22), lipids carrying with metabolism (11), etc. Interestingly, three proteins, including myosin XVB, dynein light-chain Tctex-type-1, and dynein light-chain roadblock-type-2, were found to be associated with cell motility.

**Figure 5:**
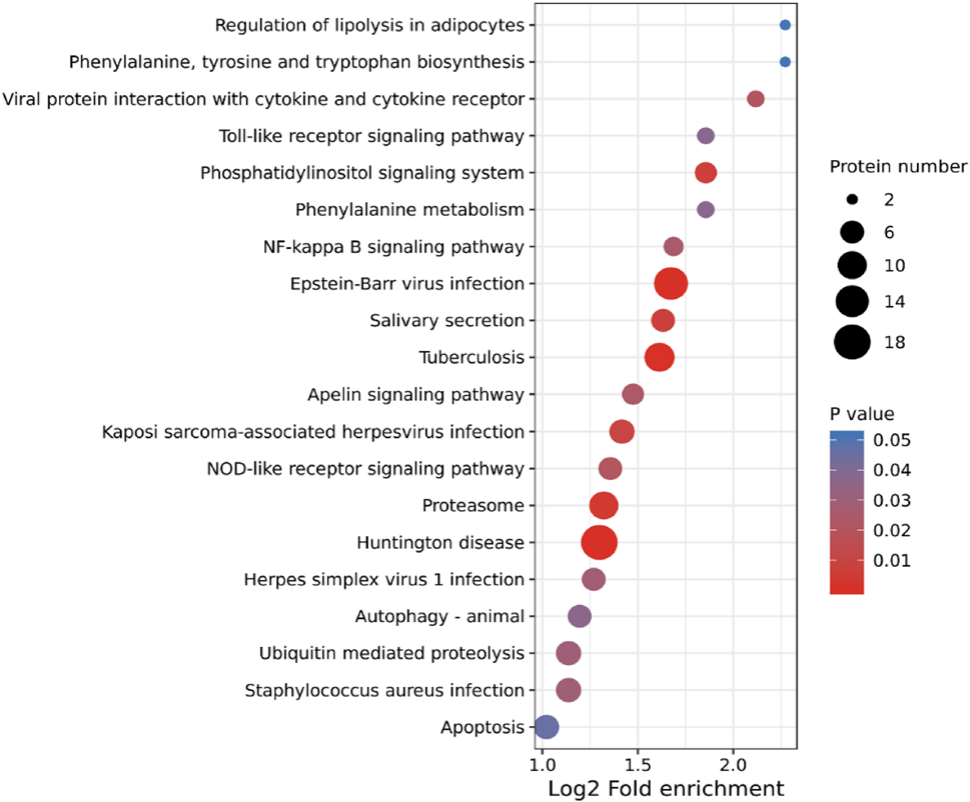
Kyoto Encyclopedia of Genes and Genomes (KEGG) pathway enrichment analysis of DAPs. The *P* value was calculated using a Fisher’s exact test. The X-axis represents the Log2 transformed ratio of DAPs compared to the identified proteins, and the Y-axis means KEGG pathway. The size of each bubble refers to the number of the DAPs. The color indicates the significance *P* value, and the size of circle indicates the number of DAPs in terms.

Subsequently, the results of the KEGG study were presented in Fig. 5., including the top 20 substantially enriched pathways. The main pathways were revealed, including the phosphatidylinositol signaling system, salivary secretion, proteasome, apoptosis, mitophagy-animal, NOD-like receptor signaling pathway, etc. Furthermore, some DAPs were detected to be potentially involved in disease or infection, such as tuberculosis, Kaposi sarcoma-associated herpesvirus infection, Huntington disease, staphylococcus aureus infection, etc.

### PRM validation

In this study, in order to confirm the accuracy of the findings acquired in relation to seminal plasma proteome, 19 DAPs were selected for the PRM analysis, including phosphoglycerate mutase, ras-related protein Rab-11B, ATP-citrate synthase, peroxiredoxin, spermadhesin-1, T complex protein-1 sub-unit alpha, programmed cell death protein 5, testis-tissue sperm binding protein Li 69n, ubiquitin-like modifier-activating enzyme 1, zonadhesin, superoxide dismutase, sperm equatorial segment protein 1, thioredoxin reductase 2 (mitochondrial), acrosin-binding protein, zona pellucida binding protein, heat shock protein family-E (Hsp10) member-1, peroxiredoxin-1, aquaporin 7, and izumo sperm-egg fusion protein 4. Based on the PRM results presented in Table 1, except spermadhesin-1, programmed cell death protein 5, heat shock protein family-E (Hsp10) member-1, and peroxiredoxin-1, the abundance of the other 15 proteins showed significant differences between the high motility group and the low motility group (*P* < 0.05). In addition, although no significant differences were found among the above mentioned four proteins, the pattern of the fold changes in all 19 selected proteins was similar with the TMT results.

**Table 1:** Validation of DAPs using parallel reaction monitoring (PRM) analysis.

Protein Accession	Protein name	Protein gene	G/B Ratio (PRM)*	G/B *P* value (PRM)	G/B Ratio (TMT)*	G/B *P* value (TMT)
A0A452EPH7	Phosphoglycerate mutase	BPGM	3.54	1.84E−02	1.64	0.0078
I1W1N4	Ras-related protein Rab-11B	RAB11B	4.28	5.70E−03	1.73	0.0033
A0A452FSK9	ATP-citrate synthase	ACLY	4.40	3.88E−02	1.52	0.0277
A0A452E5B4	Peroxiredoxin	PRDX5	2.88	3.19E−02	1.53	0.0345
A0A452DLM2	Spermadhesin-1	LOC102175091	2.84	8.60E−02	1.75	0.0221
A0A452EVX4	T-complex protein 1 subunit alpha	TCP1	2.57	3.16E−02	1.59	0.0116
A0A452DSU1	Programmed cell death protein 5	PDCD5	1.86	2.08E−01	1.53	0.0480
A0A452G6T9	Testis tissue sperm-binding protein Li 69n	PSMD14	3.72	6.53E−03	1.65	0.0002
A0A452FJ29	Ubiquitin-like modifier-activating enzyme 1	UBA1	2.78	1.23E−02	1.50	0.0005
A0A452EC76	Zonadhesin	ZAN	0.01	3.27E−03	0.35	0.0000
A0A452E294	Superoxide dismutase	SOD2	0.24	1.00E−03	0.41	0.0001
A0A452EFP3	Sperm equatorial segment protein 1	SPESP1	0.05	8.31E−05	0.51	0.0001
A0A452DL42	Thioredoxin reductase 2, mitochondrial	TXNRD2	0.24	1.48E−02	0.57	0.0001
A0A452E876	Acrosin-binding protein	ACRBP	0.19	2.95E−02	0.41	0.0009
A0A452DWD6	Zona pellucida binding protein	ZPBP	0.23	7.26E−03	0.38	0.0000
A0A452E0C3	Heat shock protein family E (Hsp10) member 1	HSPE1	0.60	7.50E−02	0.66	0.0079
A0A452E1R9	Peroxiredoxin 1	PRDX1	0.39	1.60E−01	0.61	0.0470
A0A452DNC1	Aquaporin 7	AQP7	0.22	4.31E−06	0.60	0.0001
A0A452EAI1	Izumo sperm-egg fusion protein 4	IZUMO4	0.08	6.55E−03	0.44	0.0036

“B” represented the group with low motility; “G” represented the group with high motility.

### Identification of metabolomic data

The metabolomic analysis of goat seminal plasma was performed using a non-targeted metabolomics strategy, including the positive and negative modes. In the present study, a total of 4603 metabolites have been quantified in seminal plasma derived from the two groups with high or low motility, including amino acids, peptides, fatty acids, lipids, sugars, nucleic acid, organic acids, and other metabolites. The detailed information associated with these identified metabolites, including index, mass, retention time (RT), compounds, formula, etc., was presented in Supplementary Table S3.

### Determination of differential metabolites

**Figure 6:**
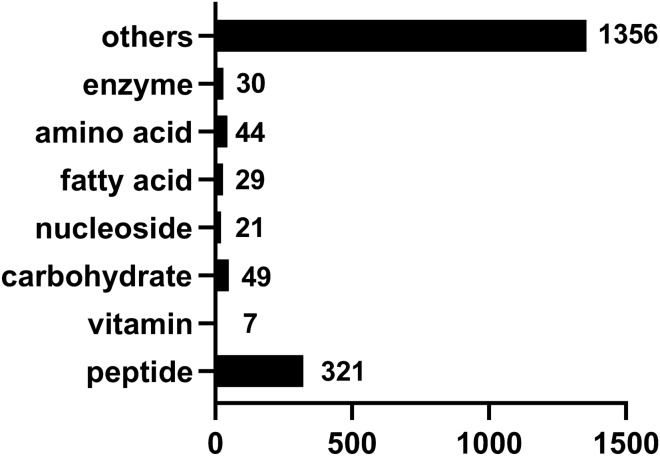
The classification of differential metabolites presented in goat seminal plasma between these two groups. The number on each column represents the number of this kind of metabolites.

Based on the Variable Importance in Projection (VIP), fold-change (FC), and *P* value, the differential metabolites between these two groups were identified. The detailed information related to these identified metabolites were presented in Supplementary Table S4. Totally, 1857 differential metabolites were identified between the high motility group and the low motility group. Among these metabolites, 999 metabolites were up-regulated in the high motility group. However, 858 metabolites were significantly enriched in the low motility group. The numbers of various compounds presented in goat seminal plasma were shown in Fig. 6.

### Bioinformatics analysis of differential metabolites

**Figure 7:**
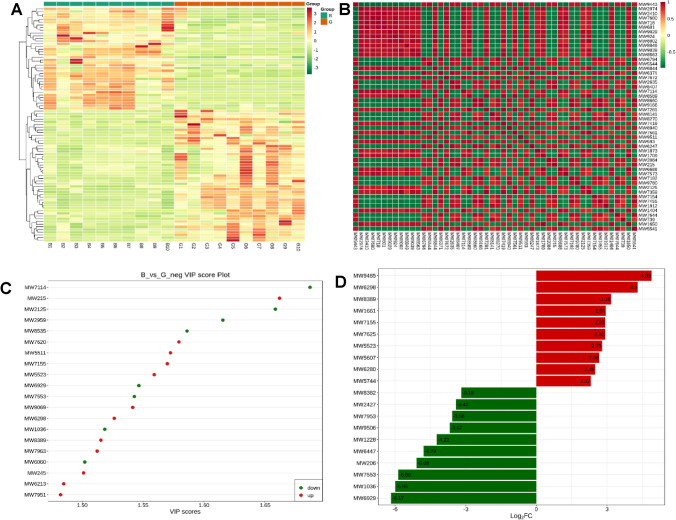
The bioinformatics analysis of differential metabolites between these two groups. The figure A represented the cluster heatmap of different metabolites. The metabolites with significant difference were normalized and clustered in this map. The X-axis represented the samples, and the Y-axis represented the differential metabolites. Red represented the highly expressed metabolites, and green represented the lowly expressed metabolites. The figure B represented the correlation results of differential metabolites analyzed by the Pearson correlation analysis method. Red color indicates strong positive correlation and green color indicates strong negative correlation. The figure C represented the top 20 differential metabolites with the largest VIP value. Abscissa represents the VIP value, and ordinate represents differential metabolite. Red represents up-regulated metabolites, and green represents down-regulated metabolites. The figure D represented the top 20 differential metabolites with the largest FC value. Abscissa represents log2FC value, and ordinate represents differential metabolites. Red represents up-regulated metabolites, and green represents down-regulated metabolites. “B” represented the group with low motility; “G” represented the group with high motility.

The cluster analysis data of the acquired differential metabolites were shown in Fig. 7. As shown in Fig. 7A., the cluster patterns between these two groups with high or low motility were opposite. The metabolites enriched in the high motility group were less abundant in the group with low motility instead. The correlation analysis results of differential metabolites were demonstrated in Fig. 7B. Here, the Pearson correlation analysis method has been used to evaluate the correlation of differential metabolites. The top 50 differential metabolites with the largest VIP value were displayed. Here, the significant correlations existed among these metabolites. TG (i-22:0/15:0/21:0) (MW9443) was negatively correlated with Trp Met (MW2974) (the correlation coefficient: −0.98), and positively correlated with (S)-N-[3-(3,4-Methylenedioxyphenyl)-2-(acetylthio) methyl-1-oxoprolyl]-(S)-alanine benzyl ester (MW5544) (the correlation coefficient: 0.96). Additionally, Hexakis (2-methyl-2-phenylpropyl) distannoxane (MW7680) was negatively correlated with MW9443 (the correlation coefficient: −0.98). However, a significantly positive correlation was found between MW7680 and DG (8:0/22:0/0:0) (MW718) (the correlation coefficient: 0.98).

In accordance to the VIP-value, the top 20 differential metabolites with largest VIP value were presented in Fig. 7C. Among these metabolites, D-Ornithine (MW7114), ostruthin (MW2125), TRIPTOPHENOLIDE (MW2959), Ornithine (MW8535), Citrulline (MW6929), Glu-Phe (MW7553), Cocaine (MW1036), and 4-formyl Indole (MW6060) were enriched in the low motility group. However, 2-Methylguanosine (MW215), Glycerophosphorylcholine (MW7620), (3S)-3,6-Diaminohexanoate (MW5511), Deoxyadenosine (MW7155), Crotonobetaine (MW5523), Saccharopine (MW9069), Adenosine (MW6298), N,N-Dimethylguanosine (MW8389), L-Threonine (MW7963), 2,3-Bis (4 hydroxyphenyl) 1,2-propanediol (MW245), 7-Methylguanosine (MW6213), and L-Prolinamide (MW7951) were more abundant in the high motility group.

In addition, based on the fold change between these two groups, the most enriched differential metabolites were shown in Fig. 7D. Thioetheramide-PC (MW9485), Adenosine (MW6298), N,N-Dimethylguanosine (MW8389), Isocitric acid (MW1661), Deoxyadenosine (MW7155), Glycocholic Acid (MW7625), Crotonobetaine (MW5523), 1-O (cis-9-Octadecenyl) 2-O-acetyl-sn glycero 3-phosphocholine (MW5607), Acetylcarnitine (MW6280), and 17alpha-Hydroxypregnenolone (MW5744) were significantly enriched in high motility group. On the other hand, N6-[(Indol-3-yl)acetyl]-L-lysine (MW8382), Pyroglutamic acid (MW2427), L-Proline (MW7953), Thr Leu Arg (MW9506), Dimethylglycine (MW1228), Arg Glu Ser Leu Glu (MW6447), 2-Keto-glutaramic acid (MW206), Glu-Phe (MW7553), Cocaine (MW1036), and Citrulline (MW6929) were more abundant in the low motility group.

### Functional annotation of differential metabolites

**Figure 8:**
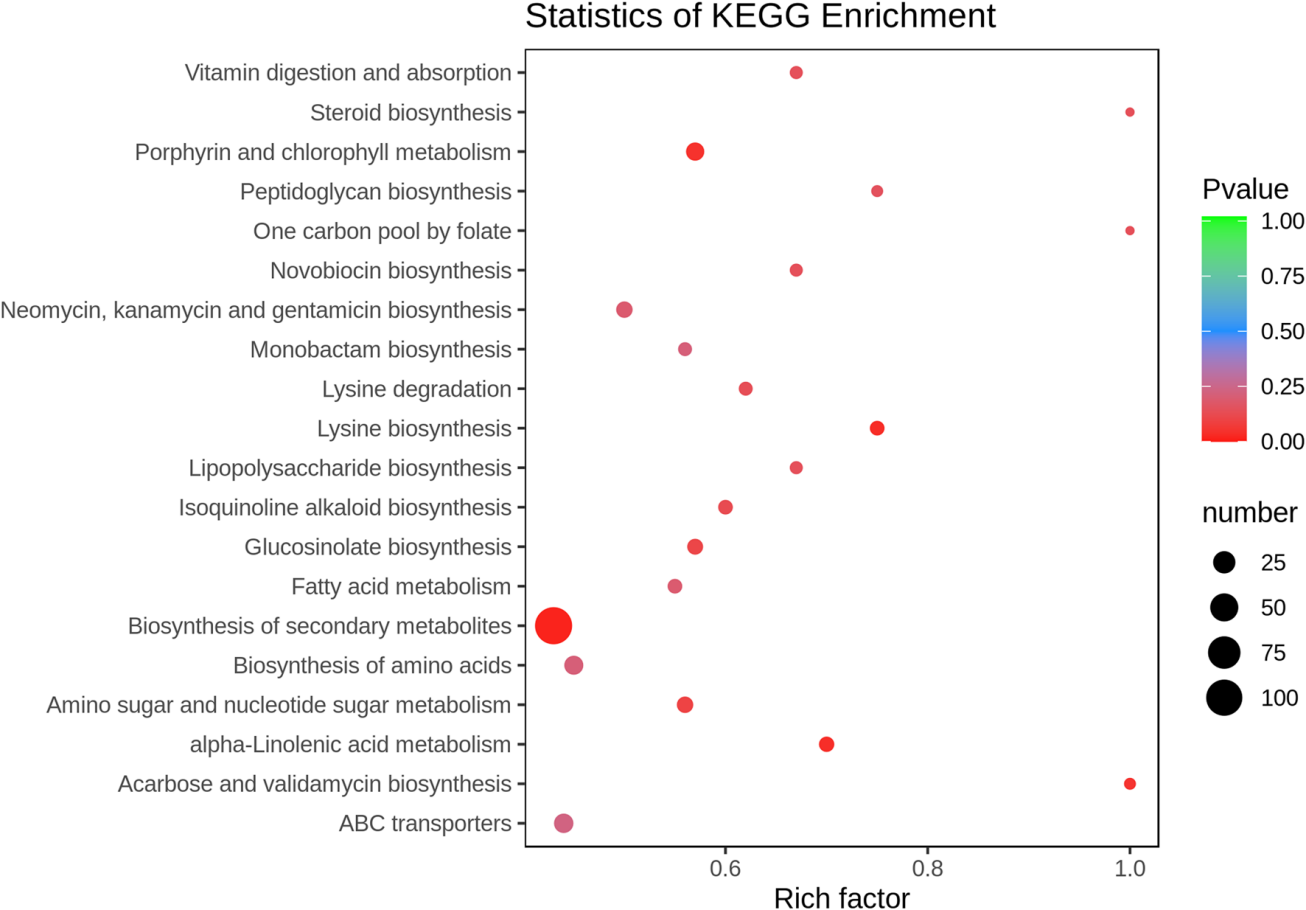
The KEGG enrichment map of differential metabolites. Abscissa represents Rich factor, and ordinate represents the pathway name. The color of the point represented the *P* value. Red indicates that the enrichment is more significant. The size of the points represents the number of metabolites involved in this pathway.

In this study, the KEGG annotation was used to identify all potential pathways which the acquired differential metabolites may be involved in. As shown in Fig. 8., most metabolites were mainly engaged on metabolic and synthetic activities, such as biosynthesis of secondary metabolites, lysine-biosynthesis, alpha linolenic-acid metabolism, porphyrin with chlorophyll metabolism, fatty acids metabolism, peptidoglycan biosynthesis, lipopolysaccharide biosynthesis, etc. The detailed information related to the KEGG annotation were included in the Supplementary Table S5.

### The correlation analysis between the obtained DAPs and differential metabolites

The correlation between the DAPs and the differential metabolites obtained in this study was analyzed using the Spearman rank sum correlation. The top 60 differential metabolites and DAPs were selected to construct the heatmap of correlation coefficients. The detailed information associated with the correlation analysis was included in the Supplementary Figure S2. Here, several typical examples with a correlation coefficient higher than 0.99 or less than −0.99 were provided. A high positive correlation between Dmx like 2 (A0A452EDH8) and 1-O-(cis-9-Octadecenyl)-2-O-acetyl-sn-glycerol-3-phosphocholine (MW5607), between phospholipase DDHD1 (A0A452G544) and Pro Pro Asp Gln Gln (MW2351), between kinesin—like protein (A0A452DNH8) and CE(5M7) (MW6801) or 2,3 –Bis (4-hydroxyphenyl)-1,2-propanediol (MW245), and between oxidoreductase HTATIP2 (A0A452FZR8) and portulacaxanthin III (MW8846) or Trp Lys Phe (MW9627) was found. On the contrary, there was a strongly negative correlation between WD repeat-containing protein 13 (A0A452EXU2) and Estra- 1,3,5 (10), 16- tetraen- 3- ol benzoate (MW1299) or Tyr Thr Gln (MW3004) or Xamoterol (MW9766) or His Ala Gln Lys (MW1539), between monocarboxylic acid transporter 1 (A0A452EWC8) and ascorbate 2- sulfate (MW6478), between WDR51B protein (A0A452G7C1) and ascorbate 2- sulfate (MW6478), and between fructose-bisphosphate aldolase (A0A452EGY9) and ascorbate 2- sulfate (MW6478). These correlation analysis results may imply the roles of seminal plasma in sperm physiological function.

## Discussion

It has been reported that sperm motility is tightly associated with the results of AI^5–7^. Furthermore, seminal plasma plays a critical role in sperm motility regulation^9–11^. Mechanisms which lead to poor sperm motility, however, are still not clear and need to be elucidated. Therefore, the relationship between seminal plasma components and sperm motility needs to be explored. In the current study, we explored the effects of the protein and metabolite components included in seminal plasma on goat sperm motility using the proteomic and metabolic methods. The high-throughput technology can act as an efficient method to classify and identify proteins and metabolites for the prediction of their potential roles in complicated biological systems^18,21^. We first established the proteome and metabolome datasets of goat seminal plasma, and then analyze potential functions and pathways in which the detected DAPs and differential metabolites may be involved. These outcomes may be helpful for further understanding of mechanisms leading to poor sperm motility.

Besides, the PRM, as a newly developed MS technology, was used to verify the TMT results in the present study. In previous studies, this technology has been used for the quantification and detection of specific proteins among biological samples^21^. It can calculate dozens of proteins simultaneously with greater quantity sensitivity and assurance as compared to those conventional methods of protein verification including western blot or immunofluorescence approach^22,23^. Based on the acquired results, a high consistency between the PRM results and the TMT results further confirmed that the proteomic data acquired in this study were accurate and believable. In addition, the percentages of sperm with intact plasma membrane among these samples used were similar, implying that the proteins and metabolites identified in seminal plasma may not be derived from leakage of sperm during the treatment.

In accordance with our study, the abundance of 445 seminal plasma-derived proteins was significantly different between the high motility group and the low motility group, and these proteins may be related to the alteration of sperm motility. Here, it should be noted that some proteins are simultaneously presented in sperm and seminal plasma. In previous studies, the similar phenomenon was also observed^24–26^. Some major proteins in sheep seminal plasma, such as ram seminal vesicle protein 14 and bodhesin 2, also appeared in plasma membrane^25^. In ring-tailed coatis, some proteins identified in sperm also appeared in their corresponding seminal plasma^24^. Furthermore, lots of proteins identified in human seminal plasma (70%) are also a part of sperm proteome^26^. Similarly, in this study, some proteins identified in seminal plasma, such as sperm lysozome-like protein 1 and ATP-citrate synthase, were also found to exist in sperm. However, it is difficult to explain this phenomenon at present. We speculated that these extracellular proteins may act as a protein reservoir and be transported into sperm through some unknown mechanisms or pathways. The functional roles of exosome can be used as an example to support this hypothesis. Exosomes are small membranous vesicles (30–150 nm) released by various cells into extracellular environment and have been proved to be presented in seminal plasma. Exosomes can transport and exchange proteins, RNAs, and lipids between cells as a means of intercellular communication^27^. So, these noncellular vesicles may act as vehicles to transport seminal plasma-derived proteins into sperm. Additionally, a recent study confirmed the transportation of seminal plasma-derived proteins to sperm plasma membrane via extracellular vesicles in multiple species^28^.

Although many proteins were detected in goat seminal plasma in this study, how to elucidate their functionality is a great challenge. After all, the reports on their functional roles were largely less than the reports in sperm. However, the studies related to sperm proteome can give me some enlightenments. The GO results showed that the DAPs primarily function in metabolic activities including the metabolism of organic substances, the cellular metabolic procedure, the main metabolic procedure, and the metabolic procedure of nitrogen compounds. The normal moving capability of sperm requires the support of ATP. Some DAPs related to ATP production were found in this study. ATP citrate synthase was more abundant in the high motility group than the low motility group. This protein is involved in ATP synthesis. In rat, reduced sperm motility and concentration were found to be induced by a reduction in the activity of ATP-citrate synthase^29^. A decrease in the level of cellular ATP and an increase in oxidative stress were also observed^29^. By contrast, in seminal plasma, the level of ATP synthase may be negatively associated with bull fertility^30^. Additionally, phosphoglycerate kinase 2 (PGK2), an isozyme that catalyzes the first step in the ATP-generating glycolytic pathway, was also up-regulated within the high motility group. This protein is also known for its important roles in sperm motility and male fertility^31^. Similarly, 6-phosphogluconate dehydrogenase was also involved in the pentose phosphate pathway and ATP production, consequently supporting motility of sperm^32^.

84 DAPs were found to be associated with response to stress. Mature sperm are extremely sensitive to environmental stimuli. Sperm lysozyme-like protein 1, as an intra-acrosomal oolemmal-binding sperm protein, was significantly down-regulated in the high motility group. This protein has been found to be involved in binding of sperm to oocyte plasma membrane during fertilization^33^. Also, the abundance of heat shock protein family E increased instead in the low motility group. In human, heat shock proteins are correlated with male fertility^34^. Furthermore, in bull, the level of sperm heat shock protein reduced significantly after the cryopreservation process, which possibly linked with reduced sperm motility, plasma membrane integrity and acrosome integrity^35^. As another specific protein presented in seminal plasma, the level of spermadhesin-1 significantly reduced in the low motility group. However, in bull, this protein was found to characterize the low-fertility phenotype after cryopreservation^30^. In another study, similar to our study, spermadhesin-1was more abundant in bulls with higher fertility^36^.

Regarding molecular function, 23 DAPs were found to function in regulation of oxidoreductase activity. Peroxiredoxin, an important antioxidant in mammalian semen, was less abundant in the low motility group. In human, the sperm suspension supplementation obtained from asthenozoospermic men efficiently enhanced sperm motility and DNA integrity through minimizing reactive oxygen levels^37^. However, in bull, the contrary results were obtained. The level of peroxiredoxin showed a negative relationship with sperm fertility^30^. Superoxide dismutase 1 plays a pivotal role in antioxidation by scavenging superoxide anions. Some studies have demonstrated that superoxide dismutase 1 is tightly associated with sperm quality, including sperm motility^38–40^. In some previous studies, superoxide dismutase was found to exist in seminal plasma, and may be associated with sperm fertility and cryotolerance^41,42^. But, in this study, superoxide dismutase 1 was found to be more abundant in the low motility group. We preliminarily hypothesize that this phenomenon may be related to a compensatory increase in goat seminal plasma with low motility.

The COG/KOG functional classification was also used to assess the potentially functional roles of the DAPs. 34 DAPs were found to be engaged on skeleton structure and function, including 11 DAPs up-regulated in the high motility group. A previous study has confirmed the vital roles of kinesin-like proteins in sperm flagella during spermatogenesis^43^. The other down-regulated DAPs, such as actin-like protein 7B, tektin-5, dynein, tubulin, etc., are well known to be involved in construction of skeleton. However, the role of kinesin-like proteins in seminal plasma still need to be elucidated. Furthermore, there is a potential link between the abundance of these three proteins (myosin XVB, dynein light chain Tctex-type 1, and dynein light chain roadblock-type 2) and motility.

To the best of our knowledge, this study is the first to conduct a comprehensive assessment of small ruminant seminal plasma metabolome, including amino acids, fatty acids, peptides, sugars, nucleosides, organic and inorganic compounds. Moreover, an association of specific seminal plasma metabolites with goat sperm motility were determined. Metabolites are derived from metabolic reactions and are involved in many biochemical pathways^44^. Moreover, metabolites have potentiality to act as biomarkers for assessment of male fertility^45–49^. Seminal plasma shows certain qualitative and quantitative variation in its biochemical composition^50^. Currently, roles of seminal plasma are still not well known. However, exposure of sperm to some metabolite-like components, such as sugars, citric acid, amino acids, can influence sperm fertility^51^. The interaction of metabolites with other molecules in the uterine environment also affects fertilization, implantation, fetal and placental developments^52^. Furthermore, some metabolites in seminal plasma, such as amino acids, peptides, sugars, fatty acids, steroids and nucleosides, are involved in some important physiological activities, influencing energy production, motility, pH control, membrane protection and metabolic activity of sperm^53–56^.

As presently evaluated by the untargeted metabolomics analysis, the major known metabolites in goat seminal plasma were defined as peptides, followed by amino acids, enzymes, carbohydrates, fatty acids, and nucleosides. 321 peptides and 44 amino acids were identified in goat seminal plasma. However, in bull, 21 metabolites were classified as amino acids, peptides, and their analogues in seminal plasma^20^. Similarly, other researchers detected 20^57^ to 23 amino acids^58^ in bull seminal plasma using the GC-MS method. In addition, a large number of amino acids were found in goat epididymal fluid^56^. Besides involvement of composing proteins^58^, amino acids have been reported to be extensively engaged on sperm biology, including protection and regulation of metabolic activity^59^. Moreover, amino acids protect sperm during cryopreservation by reducing injury caused by lipid peroxidation and free radicals^60^.

49 carbohydrates were identified in seminal plasma. Carbohydrates are essential for sperm function because these molecules are the critical components involved in energy production pathways^55^. During glycolysis, the carbohydrates included in seminal plasma, such as fructose, are required for ATP production, leading to increased respiratory activity to support sperm motility and survival^61,62^. According to a previous investigation, fructose was one of the most predominant metabolites in bull seminal plasma^20^. However, owing to the different analyzing method used, it cannot determine the real concentration of fructose in goat seminal plasma in this study. But, the concentration of fructose in the high motility group was significantly higher than that in the low motility group. The finding was similar to that reported in bull^20^. In that study, fructose was more enriched in the high fertility group as compared to the low fertility group^20^. Fructose is the primary energy source for sperm and the major carbohydrate in seminal plasma of mammals^62^. Currently, fructose has been found in seminal plasma of several species, including bull^20^, buffalo^63^, goat^64^, ram^65^, boar^66^, human^67^, and rabbit^68^. As revealed by this study, fructose is extensively involved in fundamental pathways of energy production for goat sperm. Yousef et al. suggested that a reduction in fructose concentration observed in seminal plasma of rabbits intoxicated with aluminum chloride may be one of factors leading to reduced sperm motility^68^. Therefore, it can be concluded here that reduced fructose concentration in goat seminal plasma decreases the energy supply to sperm, negatively affecting their motility.

Citric acid, with a high fold change between these two groups, was more abundant in the high motility group. Similar to our report, in bull, a significant enrichment of citric acid in seminal plasma were found in the high fertility bull^20^, implying that citric acid may be act as a potential biomarker for assessment of sperm motility. Citric acid is also presented in semen of other species, such as boar^69^, human^67^, and rabbit^70^. Citric acid is reported to be involved in pH regulation in boar semen. Furthermore, it can act as a chelator for zinc, magnesium, and calcium^69^. In human, the concentration of zinc, magnesium and calcium in seminal plasma and their chelation influence sperm metabolism, consequently affecting sperm transport, acrosome reaction, and fertilization^71^. In addition, citric acid in seminal plasma may be associated with bull fertility by potentially affecting sperm capacitation and acrosome reaction^18^.

In conclusion, this study first established the proteomic and metabolomic databanks of goat seminal plasma. The proteins and metabolites that may be involved in regulation of sperm motility were determined. There were 175 up-regulated and 274 down-regulated proteins in high motility group. The identified DAPs were primarily engaged on some essential sperm functional pathways, such as control of biological processes, metabolic processes, organization of cellular components, phosphatidylinositol signaling system, salivary secretion, proteasome, apoptosis, etc. A total of 1857 differential metabolites were identified between the high motility group and the low motility group, and 999 metabolites were up-regulated in the high motility group. Furthermore, the differential metabolites were mainly involved in the metabolic and synthetic activities. Therefore, the proteins and metabolites acquired in the present study may be helpful for us to further understand mechanisms leading to poor sperm motility.

## Materials and methods

### Ethics statement

The ethical committee of Yunnan Animal Science and Veterinary Institute (Kunming city of Yunnan province, China) has approved all experiments including animal usage in this study (201,909,006). In addition, during the whole experiment, the authors strictly complied with Regulations on the Administration of Laboratory Animals (Order-No.2 of the State Science and Technology Commission of the people's Republic of China, 1988) and Regulations on the Administration of Experimental Animals of Yunnan Province (the Standing Committee of Yunnan Provincial People's Congress 2007.10). We confirmed that all authors complied with the ARRIVE guidelines.

### Chemicals and reagents

Unless otherwise mentioned, the chemicals, reagents, and kits have been purchased from Sigma-Chemical Company (St. Louis, Mo, United States). The Andromed extender was purchased from Minitüb GmbH (Hauptstrasse 41, 84,184 Tiefenbach, Germany).

### Animals and management

In this study, the semen used was collected from a newly developed breed-*Yunshang black goats*. To collect semen, 20 bucks (2–3 years old) were used during September of 2019 (their reproductive season). Routine anthelmintic handling and vaccination against rabies and tetanus were conducted. Before the start of this study, the used bucks have been successively checked their motility six times per year during their reproductive seasons (spring and autumn) at least, in order to ensure that their sperm motility values were constant. The bucks were raised under the standardized conditions of feeding, lodging and light. The daily diet consisted of 29.5% maize, 23% soybean, 1.5% calcium monophosphate, 1% premis, 0.5% sodium-bicarbonate, 0.5% NaCl, 19% broad bean-bran, 10% alfalfa-Grass, and 15% corn-silage. The bucks had free access to salt and drink.

### Semen collection, dilution, and motility assessment

In this study, semen was collected using artificial vagina and directly transported to the laboratory within 10 min. Two successive ejaculates of one buck obtained over a 10 min period were pooled for its semen quality analysis. Instantly after collecting, we counted volume of semen and observed semen color. Mass motility was first assessed by observing the wave motion pattern of fresh undiluted semen^7,72^. However, the assessment of mass motility is subjectively carried out on the basis of the experience and knowledge of the technicians, so it is only a rough assessment. Concentrations of sperm were analyzed using Nucleo-Counter ® SP 100™ (Chemo-Metic AS, Allerød, Denmark). Following the initially assessment, quality of the used ejaculates satisfied with the criteria in the experiments were as follows: mass motility: ≥ 3.0; sperm concentration: ≥ 2500 × 10^6^ sperm/mL; normal morphology: ≥ 75%.

After the above mass motility assessment, the motility of sperm was analyzed using a computer-assisted sperm CASA system installed with the Sperm Class Analyzer (SCA) software (SCA Evolution; Microptic, Barcelona, Spain). A specific program in this software is designed for the evaluation of goat sperm. The detailed parameter setting for this program was as follows: Calibration name, 10 × ; Calibration value (μm/pixel), 0.475323; Capture method, Ph-; Grid distance (μm), 100; Analysis timeout, 15; Box size, 152; Frame rate (fps), 25; number of images, 25; Resolution, Low; Style, automatic; Minimum Area, 3μm2; Maximum Area, 70μm2; Drifting (μm/s), 0; Static (μm/s) < 10; slow-medium velocity (μm/s), 45; Rapid velocity (μm/s), 75; progressive motility (STR >), 80; connectivity (pixels), 12; VAP points (pixels), 5; VCL/VAP, VCL.

When the motility was examined, the collected semen samples were diluted using the Andromed extender to a final concentration of 20 × 10^6^ sperm/mL. 10 μl drop of sperm solution was placed on a slide and covered using a cover slip (18 mm × 18 mm). Initially, the heated plate (38 °C) with a magnification of 100 × have been installed on a phase-contrast microscope (Nikon, ECLIPSE-E200, Japan), and the progressive motility (PM, %) values were analyzed. Ten fields per drop including a total of 500 sperm has been recorded for every sample. Based on the obtained sperm motility values, the used bucks were separated into two groups with a higher (≥ 75%) or lower motility (≤ 65%).

When performing the proteomic analysis of goat seminal plasma, there are 5 bucks with higher or lower motility in each group. However, when performing the metabolomic analysis of seminal plasma, there are 10 bucks with higher or lower motility in each group. The semen from these bucks used were analyzed separately and not pooled during this whole experiment.

### Sperm plasma membrane and acrosome assessment

The hypo-osmotic swelling test (HOST) has been used to test the integrity of sperm plasma membrane as described in a previous study^73^. In brief, 20 µL of semen was incubated in 200 µL of the hypo-osmotic solution (9 g/l fructose and 4.9 g/l sodium citrate, 100 mOsm/kg) at 37 °C for 60 min. Then, 10 µL of solution was mounted on a microscope slide and covered using a cover slip. A total of 200 sperm were assessed in each time. Sperm with visible coiling tails were counted under the phase contrast microscope with a magnification of 400 × for each sample.

FITC-PSA staining together with flow cytometry was used to assess the acrosome status of goat sperm^72^. In brief, semen was diluted using the TALP buffer (95.0 mM NaCL, 3.0 mM KCL, 0.3 mM NaHPO_4_, 10.0 mM NaHCO_3_, 0.4 mM MgCL_2_·6H_2_O, 2.0 mM pyruvic acid, 5.0 mM glucose, 25 mM Na lactate, 40 mM HEPES, 3.0 mg/ml bovine serum albumin, 30.0 μg/ml gentamycin sulfate) to a fixed concentration of 10 × 10^6^ sperm/mL. Then, 200 μL of the above sample was stained using 50μL propidium iodide (PI) (50 μg/mL) and 0.5μL FITC PSA (2 mg/mL), followed by incubation in a dark and humid environment for 15 min at 37 °C. Finally, the percentages of FITC-PSA and PI stained sperm were analyzed by flow cytometry. The concentration of alive sperm with intact acrosome and plasma membrane were identified as PI and FITC-PSA negative.

A FacStar-plus flow cytometer (FAC SCalibur, Becton–Dickinson and Co., Franklin Lakes, NJ, USA) was used to perform the flow cytometry analysis. The green fluorescence emitted from FITC-PSA were detected on the FL1 photodetector (530/30BP-filter). The red fluorescence generated from PI was observed on the FL2 photodetector (670LP-filter). The Ar ion blue laser was used to excite those fluorochromes (488 nm). The fluorescence information was shown in the logarithmic mode using the Cell-Quest Pro-3.1 program (BD-Biosciences). According to the guideline of International Society for the Advancement of Cytometry (ISAC), the data was obtained from 100,000 events for further study using the Cell-Quest program (Becton Dickinson).

### Seminal plasma exaction and purification

The seminal plasma exaction process was defined in a previous report^74^. It should be noted that seminal plasma was isolated and purified from the same ejaculate assessed by the CASA. In brief, following semen collection and assessment, seminal plasma was extracted separately from sperm cells via centrifugation at 10,000 × g for 10 min in a microfuge at 4 °C. Then, the supernatants were gently collected and centrifuged again at the same condition. The collected seminal plasma was further filtered via a 0.22 μm Millipore filter (Millipore). The seminal plasma samples were preserved at −80 °C for the proteomics and metabolomic analysis.

### Protein extraction and trypsin digestion

The protein extraction process has been described in a previous study^22^. In brief, before the extraction of total proteins in seminal plasma, all samples were initially sonicated for three times using ice, applying the highly intensity ultrasonic-processor (Scientz) in the lysis buffer (8 M urea, 1% protease inhibitor cocktail). The supernatants were collected after centrifugation at 12,000 g at 4 °C for 10 min, and the protein concentrations were measured using the BCA kit as instructed by the manufacturer.

The protein mixture was reduced by 5 mM dithiothreitol at 56 °C for 30 min and alkylated using 11 mM iodoacetamide for 15 min at room temperature in darkness for absorption. The urea concentration in the protein samples were diluted to less than 2 M applying 100 mM triethylammonium bicarbonate. After the above treatments, trypsin was applied for the first digestion overnight at a trypsin to protein mass ratio (1: 50), and then for the second digestion for 4 h at a trypsin to protein mass ratio (1: 100). 200 ug protein of each sample were taken to be digested.

### TMT labeling, HPLC fractionation, and LC–MS/MS analysis

Then, 50 ug protein of each sample was used for TMT labeling. The peptides were desalinated through the Strata X-C18 SPE column (Phenomenex) and vacuum dried, following digestion with trypsin. Peptides were reassembled into 0.5 M triethylammonium bicarbonate and operated for the 10-PLEX TMT package according to the instructions of manufacture for the TMT/iTRAQ-kit. In short, one unit of the TMT/iTRAQ mixture was thawed and reassembled into 24 μl acetonitrile (defined as the volume of mixture needed to mark of 100 μg proteins). The peptide solutions were incubated for 2 h at room temperature, pooled, desalted, and dried through vacuum centrifugation.

Using an Agilent-300 Extend C18 column (5 μm particles, 4.6 mm ID, 250 mm length), the samples were fractionated into various fractions through the high-pH reverse phase HPLC. In brief, peptides were initially separated in 10 mM ammonium-bicarbonate (pH-10) for 80 min into 80 fractions with gradient of 2% to 60% acetonitrile. Later, the peptides were combined into 9 fractions and dried by vacuum centrifugation.

The tryptic peptides were dissolved in the solvent-A (0.1% formic-acid, 2% acetonitrile), and straightly loaded into a home-made reversed phase analytical column (20 cm length, 100 μm i.d.). The gradient was comprised of an increasing from 6 to 22% solvent-B (0.1% formic acid in 90% acetonitrile) during 38 min, 22% to 32% in 14 min, and an increase to 80% for 4 min, then maintaining at 80% for the last 4 min. All processes were operated at stable flow rate of 450 nL/min using the EASY-nLC 1200 UPLC system.

After HPLC, 5 ug peptide of each fraction was used for the MS analysis. The peptides were subjected to the NSI sources in Q-ExactiveTM HF X (Thermo), followed by the tandem-mass spectrometry (MS/MS) together online with the UPLC. The applied electrospray tension was 2.0 kV. The m/z assay size for a complete scan was 350 to 1600, and the Orbitrap detected the whole peptides at resolution of 120, 000. The peptides have been chosen for MS/MS with the NCE setting at 28. The fragments were identified at a resolution of 30,000 in the Orbitrap. A data dependent process that exchanged from one MS-scan to 20 MS/MS dynamic exclusion scans using 30.0 s. Automatic gain-control (AGC) was fixed at 1E5. The first set mass was fixed at 100 m/z.

### Bioinformatics analysis of proteomic data

The MS/MS data was analyzed applying the explore engine Max-Quant (v-1.5.2.8). In Capra aegagrus hircus database concatenated using the reversed decoy database, the tandem mass spectra were detected. Trypsin/P has been determined as the cleavage enzyme which allows up to 2 lacking cleavages. The parameters of searching cutoff value of mass spectrometry were set as the following: first search peptide tolerance, 20 ppm; main search peptide tolerance, 4.5 ppm; Max missed cleavages, 2; Min ratio count, 2 unique + razor Peptides for quantification; Min peptide length, 7; PSM FDR/Protein FDR, 0.01; Min score for modified peptides, 40; Min score for recalibration, 70; when bioinformatic quantification, unique peptide >  = 1. The TMT 10-PLEX was chosen for the quantification process. In MaxQuant, the parameters were fixed as the default values. The *P* values were calculated using the t-test of the two-sample two-tailed Student. Proteins with fold change of > 1.50 and *P* value < 0.05 were identified as up-regulated DAPs between the high motility group and the low motility group. However, the proteins with fold change of < 0.667 and *P* value < 0.05 were identified as the down-regulated proteins.

The Gene Ontology (GO) annotation was performed based on the UniProt GOA database (http://www.ebi.ac.uk/GOA/). At first, the IDs of identified proteins were transformed to UniProt-IDs, mapping to GO IDs with the protein IDs. Unless some identified proteins can be annotated by the UniProt-GOA database, the InterProScan will be applied to annotate protein’s GO functionality using the protein sequence alignment procedure. Based on the UniProt-GOA database, the DAPs were classified into three types: biological procedure, cell compartment, and molecular functions. For each type, two tailed Fisher’s exact-test was used to test the enrichment of the DAPs against all identified proteins. The GO with a modified *P* value < 0.05 was considered significantly. In addition, the information related to subcellular localization of the obtained DAPs was inferred with Wolfpsort (http://www.genscript.com/psort/wolfpsort.html).

The KEGG online service tools KAAS (http://www.genome.jp/kaas-bin/kaas) was used to identify pathways that the obtained DAPs are enriched. Firstly, KAAS was applied to annotate the KEGG database description of the identified proteins. Then, the annotation results were mapped on the KEGG pathway database applying the KEGG online service tools KEGG mapping. Based on the KEGG database, the two tailed Fisher’s exact-test was used to detect the enriched channels to test the enrichment of DAPs against the entire detected proteins. The pathway was considered significantly with a corrected *P* value < 0.05. According to the KEGG website, these pathways were specified into hierarchical groups^75^.

The analysis of protein domain was conducted applying the InterPro domain database (http:/www.ebi.ac.uk/interpro/). For the identified proteins in each category, the InterPro (resources that allows functional evaluation of protein sequencing by identifying proteins in various groups and estimating the existence of domains and major locations) database have been scanned, and two tailed Fisher’s exact-test has been applied to analyze the enrichment of DAPs against those identified proteins. Proteins domain with a corrected *P* value < 0.05 was thought significant.

### Parallel reaction monitoring (PRM) validation

The seminal plasma separation and total protein exaction were the same as the above procedure. The digested peptides were submitted to the PRM analysis. PRM is a newly developed approach to verify proteins using the quadrupole-Orbitrap mass spectrometer^21,76^. In brief, the tryptic peptides were mixed in the solvent A and eluted in a reversed phase analytical column using the gradient solvent B (6–25% over 40 min, 25–35% over 12 min, 80% over 4 min, and 80% over the last 4 min) at a rate of 500 nL/min. The peptides measured (1.5 mg per sample) were analyzed with an online Q Exactive™ plus the Orbitrap mass spectrometer (ThermoFisher Scientific, Waltham, MA, USA) coupled with the UPLC. The applied electrospray tension was 2.2 kV. The full MS scans (400–960 m/z) were obtained at a resolution of 70,000 using AGC of 3E6 and a highest injection time (MIT) of 50 ms. A data independent protocol (one MS scan followed by 20 MS/MS scans) was used for the MS/MS scans with the following parameters: resolution, 17,500; NEC, 27; AGC, 1E5; MIT, 120 ms; insulation window, 1.6 m/z. The PRM data was analyzed using the Skyline 3.6. The results were quantified for every peptide, and the DAPs detected were screened and compared with the MS data derived from the TMT.

### Seminal plasma metabolite exaction

In brief, the collected seminal plasma samples were first warmed on ice. The samples were vortexed for 30 s to ensure complete mixing. Then, 3 volumes of ice-cold methanol were added to 1 volume of seminal plasma, followed by vortexing for 3 min. The mixture was further centrifuged at 12,000 g for 10 min at 4 °C. The supernatant was collected and centrifuged at 12,000 g for 5 min at 4 °C again. After filtration through a 0.22 µm filter membrane, the supernatants were transferred into the injection bottles. Finally, the samples were preserved at −80 °C prior to the LC–MS/MS analysis. In addition, the pooled QC samples were simultaneously prepared by mixing 10 μL of each exacted mixture.

### HPLC

The exacted seminal plasma samples were analyzed using the LC–ESI–MS/MS system (UPLC, Shim-pack UFLC SHIMADZU CBM A system, https://www.shimadzu.com/; MS, QTRAP 6500 + System, https://sciex.com/). The analytical parameters were set as following: UPLC: column, Waters ACQUITY UPLC HSS T3 C18 (1.8 µm, 2.1 mm × 100 mm); the column temperature, 35 oC; the flow rate, 0.3 mL/min; the injection volume, 1 μL; the solvent system, water (0.01% methanolic acid): acetonitrile; the gradient program of positive ion, 95:5 (V/V) at 0 min, 79:21 (V/V) at 3.0 min, 50:50 (V/V) at 5.0 min, 30:70 (V/V) at 9.0 min, 5:95 (V/V) at 10.0 min, and 95:5 (V/V) at 14.0 min; the gradient program of negative ion, 95:5 (V/V) at 0 min, 79:21 (V/V) at 3.0 min, 50:50 (V/V) at 5.0 min, 30:70 (V/V) at 9.0 min, 5:95 (V/V) at 10.0 min, and 95:5 (V/V) at 14.0 min.

### ESI-QTRAP-MS/MS

The LIT and triple quadrupole scans were acquired on a triple quadrupole-linear ion trap mass spectrometer (QTRAP) (QTRAP 6500 + LC–MS/MS System) installed with an ESI Turbo Ion-Spray interface and controlled by the Analyst 1.6.3 software (Sciex). The QTRAP was operated in both positive and negative ion modes. The parameters of the ESI source operation were set as the following: the source temperature: 500 °C; the ion spray voltage (IS): 5500 V (positive) and −4500 V (negative); the ion source gas I (GSI): 55.0 psi; the gas II (GSII): 60.0 psi; the curtain gas (CUR): 25.0 psi; the collision gas (CAD): high. The instrument tuning and mass calibration were performed in 10 and 100 μmol/L polypropylene glycol solutions using the QQQ and LIT modes, respectively. A specific set of MRM transitions were monitored during each period based on the metabolites eluted within this period.

### Metabolomics data analysis

The original data files acquired by the LC–MS analysis were first converted into mzML format using the ProteoWizard software. Peak extraction, alignment, and retention time correction were performed by the XCMS program. The “SVR” method was used to correct the peak area. The peaks were filtered in accordance with a deletion rate > 50% in each group of samples. After the above treatments, the identified metabolite information was obtained by searching the laboratory’s self-built database, the public database (Metlin), and metDNA. Finally, the statistical analysis was carried out by the R program. The statistical analysis includes the univariate analysis and the multivariate analysis. The univariate statistical analysis was performed using Student’s t-test and variance multiple analysis. The multivariate statistical analysis was carried out using these approaches including principal component analysis (PCA), partial least squares discriminant analysis (PLS-DA), and orthogonal partial least squares discriminant analysis (OPLS-DA).

### Statistical analysis

The data associated with motility, plasma membrane and acrosome integrity of goat sperm were statistically analyzed by T-test using the JMP10.0 software (SAS Institute Inc., Cory, NC, USA). Data normality and homogeneity of variances were verified using the Shapiro–Wilk normality tests and Levene’s tests, respectively. The data were presented as means ± SEM. It was thought that the data with value of *P* < *0.05* or *P* < *0.01* was statistically significant.

## Supplementary Information

Supplementary Information 1.

Supplementary Information 2.

Supplementary Information 3.

Supplementary Information 4.

Supplementary Information 5.

Supplementary Information 6.

Supplementary Information 7.

Supplementary Information 8.

Supplementary Information 9.
