# Supplementary material for: The characteristics of proteome and metabolome associated with contrasting sperm motility in goat seminal plasma
Source: Sci Rep. 2021 Jul 30;11:15562. doi: 10.1038/s41598-021-95138-9 (PMC8324791; doi:10.1038/s41598-021-95138-9)
Supplement: Supplementary file 1 — Supplementary Information 1. [file 41598_2021_95138_MOESM1_ESM.docx]

**The characteristics of proteome and metabolome associated with** **contrasting sperm motility in goat seminal plasma**

Baoyu Jia^a,1^, Jiachong Liang^b,c1^, Chunrong Lv^b,c^, Sameeullah Memon^b^, Yi Fang^d^, Guoquan Wu^b,c^**, Guobo Quan^b,c^*

a College of Veterinary Medicine, Yunnan Agricultural University, Kunming city, Yunnan province, China

b Yunnan Animal Science and Veterinary Institute, Jindian, Panlong County, Kunming City, Yunnan province, China

c Yunnan Provincial Engineering Laboratory of Animal Genetic Resource Conservation and Germplasm Enhancement, Jindian, Panlong County, Kunming City, Yunnan province, China

d Jilin Provincial Key Laboratory of Grassland Farming, Northeast Institute of Geography and Agoecology, Chinese Academy of Sciences, Changchun city, Jilin provinve, China

* Corresponding author.

** Corresponding author.

1. mail addresses: waltq20020109@163.com (G.-B. Quan), wuguoquan1982@163.com (G.-Q. Wu).

^1^ These authors contributed equally to this work.
