# Supplementary figures and images for: The characteristics of proteome and metabolome associated with contrasting sperm motility in goat seminal plasma
Source: Sci Rep. 2021 Jul 30;11:15562. doi: 10.1038/s41598-021-95138-9 (PMC8324791; doi:10.1038/s41598-021-95138-9)

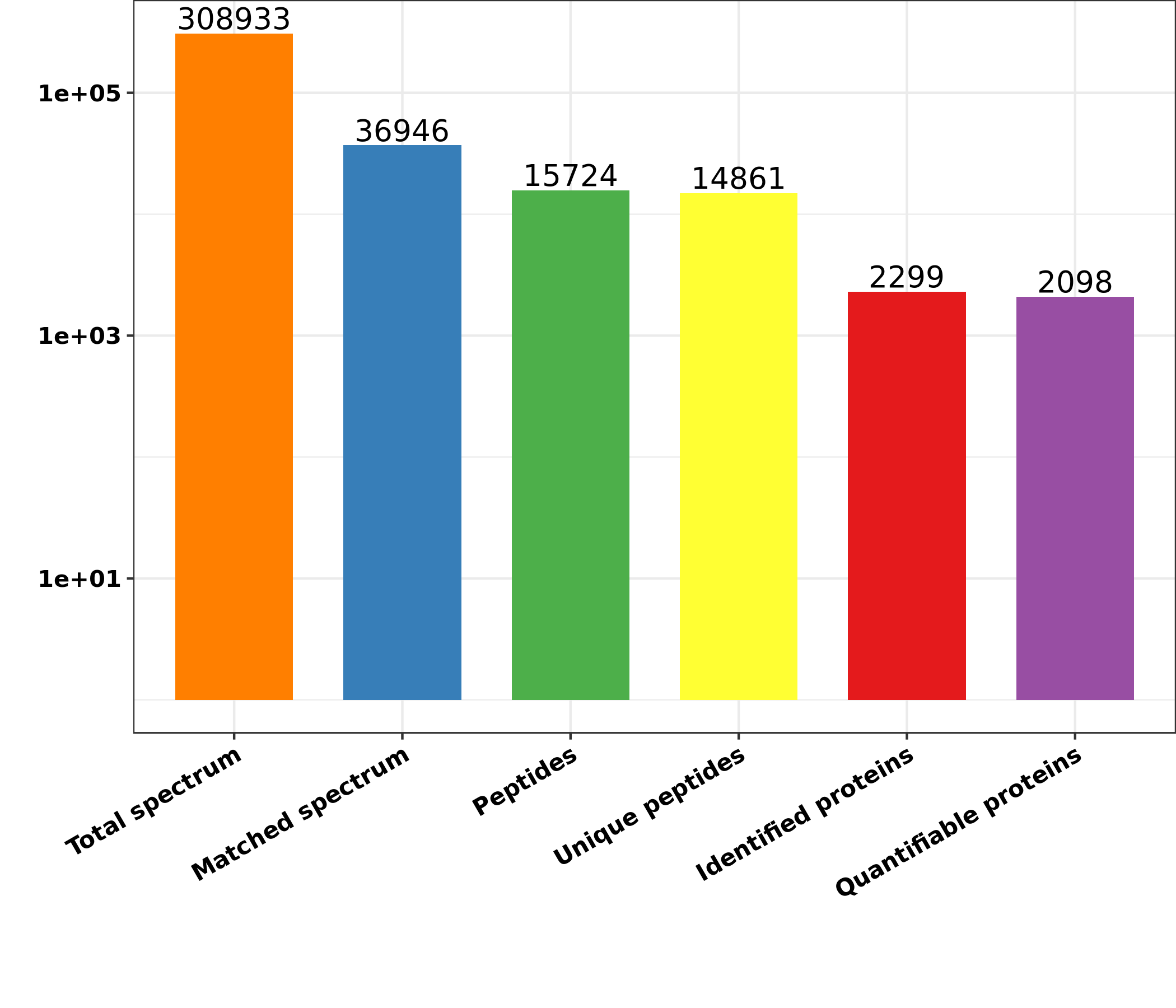

Supplement: Supplementary file 2 — Supplementary Information 2. [file 41598_2021_95138_MOESM2_ESM.png]

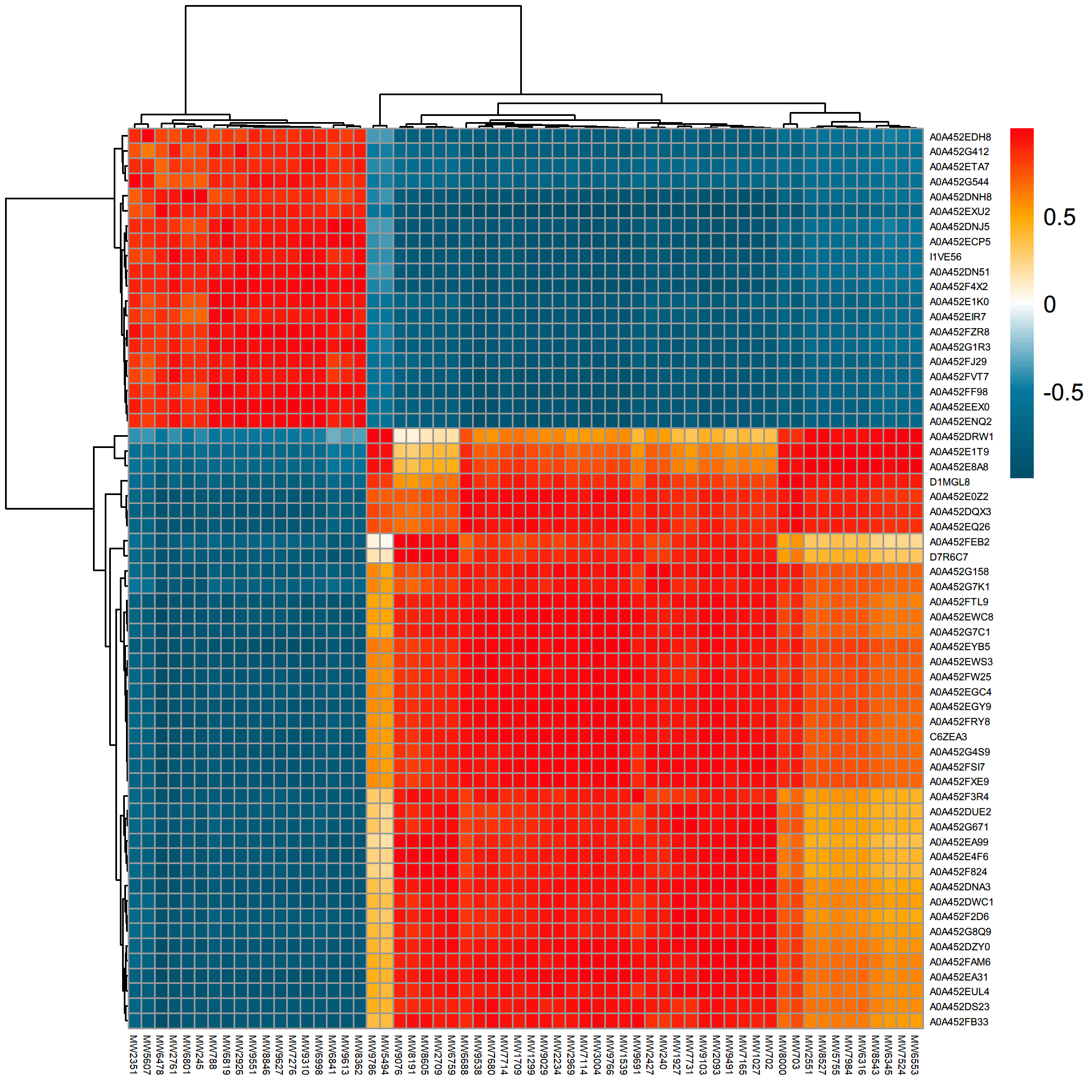

Supplement: Supplementary file 3 — Supplementary Information 3. [file 41598_2021_95138_MOESM3_ESM.png]
