# Supplementary material for: The characteristics of proteome and metabolome associated with contrasting sperm motility in goat seminal plasma
Source: Sci Rep. 2021 Jul 30;11:15562. doi: 10.1038/s41598-021-95138-9 (PMC8324791; doi:10.1038/s41598-021-95138-9)
Supplement: Supplementary file 9 — Supplementary Information 9. [file 41598_2021_95138_MOESM9_ESM.docx]

**The legends of supplementary figures and tables**

Supplementary Figure S1. The basic proteomic information of goat seminal plasma.

Supplementary Figure S2. The correlation between the DAPs and the differential metabolites. The horizontal axis represented the clustering of the differential metabolites, and the vertical axis represented the clustering of the DAPs. The shorter the distance between these cluster branches is, the higher the similarity is. Red color represented the positive correlation between the DAPs and the differential metabolites. On the contrary, blue color represented the negative correlation.

Supplementary Table S1. The basic information on the protein profile of goat seminal plasma.

Supplementary Table S2. The information associated with the differentially expressed proteins identified in goat seminal plasma with high or low motility.

Supplementary Table S3. The information associated with the identified metabolites in goat seminal plasma.

Supplementary Table S4. The information related to the identified metabolites in goat seminal plasma with high or low motility. The differential metabolites were identified based on the Variable Importance in Projection (VIP), fold-change (FC), and *P-*value.

Supplementary Table S5. The detailed information related to the KEGG annotation of the identified differential metabolites.
